# Mathematical Analysis and Treatment for a Delayed Hepatitis B Viral Infection Model with the Adaptive Immune Response and DNA-Containing Capsids

**DOI:** 10.3390/ht7040035

**Published:** 2018-11-19

**Authors:** Jaouad Danane, Karam Allali

**Affiliations:** Laboratory of Mathematics and Applications, Faculty of Sciences and Technologies, University Hassan II of Casablanca, P.O. Box 146, 20650 Mohammedia, Morocco; jaouaddanane@gmail.com

**Keywords:** HBV virus, abaptive immune response, treatment, numerical simulation

## Abstract

We model the transmission of the hepatitis B virus (HBV) by six differential equations that represent the reactions between HBV with DNA-containing capsids, the hepatocytes, the antibodies and the cytotoxic T-lymphocyte (CTL) cells. The intracellular delay and treatment are integrated into the model. The existence of the optimal control pair is supported and the characterization of this pair is given by the Pontryagin’s minimum principle. Note that one of them describes the effectiveness of medical treatment in restraining viral production, while the second stands for the success of drug treatment in blocking new infections. Using the finite difference approximation, the optimality system is derived and solved numerically. Finally, the numerical simulations are illustrated in order to determine the role of optimal treatment in preventing viral replication.

## 1. Introduction

Hepatitis B virus (HBV) infects hepatocytes and causes approximately one million deaths annually [1]. With more than 257 million infected persons; HBV is considered a global public health problem [2]. This dangerous epidemic can be easily transmitted through contact with infected body fluids [3]. After transmission of the infection, HBV can cause acute or chronic illness [4]. Many mathematical models have been developed in order to study and model the dynamics of this serious viral infection [5,6,7,8]. All these models include the interaction between HBV and both the healthy and infected liver cells. The models which include the adaptive immune response in fighting the free viruses and in reducing the infected cells have been studied [9,10,11,12]. This adaptive immunity is represented by cytotoxic T-lymphocytes (CTL) and antibody immune responses. The mathematical analysis of HBV viral infection with HBV DNA-containing capsids was determined [13,14,15,16,17]. It has also been noted that the infected liver cells release the HBV DNA-containing capsids under the form of mature viruses after being enveloped by both cellular membrane lipids and viral envelope proteins [18,19]. More recently, the optimal control of HBV infection including HBV DNA-containing capsids and CTL immune response was studied [17]. In this paper, we are interested in the same problem, but we will introduce antibodies into this model. This work is motivated by the role of antibodies in reducing the viral infection severity [20,21,22]; thus it will be important to consider such an interesting element in studying the HBV viral dynamics. The model under consideration will be stated under the form of the following nonlinear system of six differential equations:

(1) $$\left\{\begin{array}{c} \;\frac{dX}{dt}=s-\mu X\left(t\right)-k\left(1-\eta_{1}\right)X\left(t\right)Y\left(t\right),\\ \;\frac{dY}{dt}=e^{-\lambda \tau }k\left(1-\eta_{1}\right)X\left(t-\tau \right)V\left(t-\tau \right)-\delta Y\left(t\right)-pY\left(t\right)Z\left(t\right),\\ \;\frac{dD}{dt}=\left(1-\eta_{2}\right)aY\left(t\right)-\beta D\left(t\right)-\delta D\left(t\right),\\ \;\frac{dV}{dt}=\beta D\left(t\right)-uV\left(t\right)-qV\left(t\right)W\left(t\right),\\ \;\frac{dW}{dt}=gV\left(t\right)W\left(t\right)-hW\left(t\right),\\ \;\frac{dZ}{dt}=cY\left(t\right)Z\left(t\right)-bZ\left(t\right). \end{array}\right.$$

The uninfected cells *X* are produced with an average *s*, die with a rate μ, and become infected by the virus with a rate *k*. Infected cells *Y* die with an average δ and are killed by the CTL immune system with an average *p*. The constant λ is the death average of infected but still not virus-producing cells. The intracellular delay, τ, stands for the time needed for infected cells to produce new viruses after viral entry. The term $e^{-\lambda \tau }$ is the probability of surviving between t−τ and *t*. The capsids *D* are produced with a rate *a*, they are transmitted to blood with a rate β and die with a rate δ. The free viruses *V* grow with a rate β, decay at a rate *u* and are neutralized by antibodies with a rate *q*. Antibodies *W* expand in response to free virus with a rate *g* and decay at a rate *h*. CTLs *Z* develop in response to viral antigen derived from infected cells with a rate *c* and decay in the absence of antigenic stimulation with a rate *b*. Finally, $\eta_{1}$ and $\eta_{2}$ denote the efficiency of pegylated interferon (PEG IFN) and lamivudine (LMV) drugs, respectively. It is noteworthy to mention that the main function of the PEG IFN drug is to block new infections of the healthy hepatocytes in the liver, while the prime role of the second drug, LMV, is to stop viral production [16,17].

The organization of this paper is as follows. The next section is concerned to the analysis of the model. Section 3 is devoted to an optimization analysis of our suggested viral infection model. In Section 4, we construct an appropriate numerical algorithm and show some numerical simulations. The last section concludes the work.

## 2. Analysis of the Model

### 2.1. Non-Negativity and Boundedness of Solutions

The model (1) represents a system of six delayed differential equations. For such kind of problems, initial functions have to be stated and the functional framework needs to be specified. Let $X\;=\;C(\left[-\tau ,0\right];R^{6})$ be the Banach space of continuous mapping from [−τ,0] to $R^{6}$ supplied by the sup-norm $∥\varphi ∥=\underset{-\tau \leq t\leq 0}{\sup}\varphi \left(t\right)$. The initial functions of the problem verify the following:

(2) (X(θ),Y(θ),D(θ),V(θ),W(θ),Z(θ))∈X.

Also, for biological reasons, these six initial functions X(θ), Y(θ), D(θ), V(θ), W(θ) and Z(θ) have to be non-negative:

(3) X(θ)≥0,Y(θ)≥0,D(θ)≥0,V(θ)≥0,W(θ)≥0,Z(θ)≥0,forθ∈[−τ,0].

We have the following result about the boundedness and the positivity of any solutions of the system (1):

**Theorem** **1.** *For any initial functions X(θ), Y(θ), D(θ), V(θ), W(θ) and Z(θ)) verifying* (2) *and* (3), *the system* (1) *has an unique solution; moreover, this solution is non-negative and bounded for all t≥0.*

**Proof.**  By the classical theory of the functional differential equations (see for instance [23], and the references therein), we know that there is a unique local solution (H(t),I(t),D(t),V(t),W(t),Z(t)) to system (1) in $\left[0,t_{m}\right)$, where $t_{m}$ is a finite number.

By using the system (1), we have

$$X\left(t\right)=e^{-\int_{0}^{t}\left(\mu +k\left(1-\eta_{1}\right)V\left(\xi \right)\right)d\xi }\left(X\left(0\right)+\int_{0}^{t}se^{\int_{0}^{\eta }\left(\mu +k\left(1-\eta_{1}\right)V\left(\xi \right)\right)d\xi }d\eta \right),$$

$$Y\left(t\right)=e^{-\int_{0}^{t}\left(\delta +pZ\left(\xi \right)\right)d\xi }\left(Y\left(0\right)+\int_{0}^{t}\beta e^{-\lambda \tau }\left(1-\eta_{1}\right)V\left(\eta -\tau \right)X\left(\eta -\tau \right)e^{\int_{0}^{\eta }\left(a+pZ\left(\xi \right)\right)d\xi }d\eta \right)$$

$$D\left(t\right)=e^{-(\delta +\beta )t}\left(D\left(0\right)+\int_{0}^{t}\left(1-\eta_{2}\right)aY\left(\eta \right)e^{(\delta +\beta )\eta }d\eta \right),$$

$$V\left(t\right)=e^{-\int_{0}^{t}\left(u+qW\left(\xi \right)\right)d\xi }\left(V\left(0\right)+\int_{0}^{t}\beta D\left(\eta \right)e^{-\int_{0}^{\eta }\left(u+qW\left(\xi \right)\right)d\xi }d\eta \right),$$

$$W\left(t\right)=W\left(0\right)e^{\int_{0}^{t}\left(gV\left(\xi \right)-h\right)d\xi }$$

and

$$Z\left(t\right)=Z\left(0\right)e^{\int_{0}^{t}\left(cY\left(\xi \right)-b\right)d\xi },$$

By recursive argument we get that X(t)≥0,Y(t)≥0,D(t)≥0,V(t)≥0,W(t)≥0, Z(t)≥0, forallt≥0, this proves the positivity of solutions in $t\in [0,t_{m})$.

For the boundedness result of all the solutions, we will consider the following functional:

$$\begin{array}{cc} H(t) & =cge^{-\lambda \tau }X\left(t\right)+cgY\left(t+\tau \right)+\frac{\delta cg}{2a}D\left(t+\tau \right)\\ & +\frac{\delta cg}{2a}V\left(t+\tau \right)+\frac{\delta cq}{2a}W\left(t+\tau \right)+gpz\left(t+\tau \right). \end{array}$$

Therefore, when we use (1), we have

$$\begin{array}{cc} \frac{dH(t)}{dt}= & cge^{-\lambda \tau }\left(s-\mu X\left(t\right)-\beta \left(1-\eta_{1}\right)V\left(t\right)X\left(t\right)\right)\\ & +cg\left(ke^{-\lambda \tau }\left(1-\eta_{1}\right)V\left(t\right)X\left(t\right)-\delta Y\left(t+\tau \right)-pY\left(t+\tau \right)Z\left(t+\tau \right)\right)\\ & +\frac{\delta cg}{2a}\left(\left(1-\eta_{2}\right)aY\left(t+\tau \right)-\beta D\left(t+\tau \right)-\delta D\left(t+\tau \right)\right)\\ & +\frac{\delta cg}{2a}\left(\beta D(t+\tau )-uV(t+\tau )-qW(t+\tau )V(t+\tau )\right)\\ & +\frac{\delta cq}{2a}\left(gW(t+\tau )V(t+\tau )-hW(t+\tau )\right)\\ & +pg\left(cY(t+\tau )Z(t+\tau )-bZ(t+\tau )\right), \end{array}$$

because of the fact $\eta_{2}\in \left[0,1\right]$, we have $1-\eta_{2}\leq 1$, from which, it follows

$$\begin{array}{cc} \frac{dH(t)}{dt}\leq & scge^{-\lambda \tau }-cge^{-\lambda \tau }\mu X\left(t\right)-cg\frac{\delta }{2}Y\left(t+\tau \right)-\frac{\delta cg}{2a}\delta D\left(t+\tau \right)\\ & -\frac{\delta cg}{2a}uV\left(t+\tau \right)-\frac{\delta cq}{2a}hW\left(t+\tau \right)-pbZ\left(t+\tau \right). \end{array}$$

Assuming that $\rho =min(\mu ,\frac{\delta }{2},u,h,b)$, we obtain

$$\frac{dH(t)}{dt}\leq scge^{-\lambda \tau }-\rho H\left(t\right),$$

then,

$$H\left(t\right)\leq H\left(0\right)e^{-\rho t}+\frac{scge^{-\lambda \tau }}{\rho }\left(1-e^{-\rho t}\right),$$

this shows that H(t) is bounded, and so are the other functions X(t), Y(t), D(t), V(t), W(t) and Z(t). Therefore, every local solution can be prolonged up to any time $t_{m}>0$, which means that the solution exists globally. ☐

### 2.2. Steady States

By simple calculation the system (1) has the following disease free equilibrium

$$E_{f}=\left(\frac{s}{\mu },0,0,0,0,0\right).$$

Indeed, the system (1) has four steady states other than $E_{f}$:

$$E_{1}=\left(X_{1},Y_{1},D_{1},V_{1},0,0\right),$$

where

(4) $$\begin{array}{c} X_{1}=\frac{\delta u(\beta +\delta )}{ak\beta e^{-\lambda \tau }},Y_{1}=\frac{sak\beta e^{-\lambda \tau }-\mu \delta u\beta -\mu \delta^{2}u}{ak\delta \beta },\\ D_{1}=\frac{sak\beta e^{-\lambda \tau }-\mu \delta u\beta -\mu \delta^{2}u}{k\delta \beta (\beta +\delta )},V_{1}=\frac{sak\beta e^{-\lambda \tau }-\mu \delta u\beta -\mu \delta^{2}u}{k\delta \beta u(\beta +\delta )}, \end{array}$$

$$E_{2}=\left(X_{2},Y_{2},D_{2},V_{2},0,Z_{2}\right),$$

where

(5) $$\begin{array}{cccc} X_{2}=\frac{scu(\delta +\beta )}{ab\beta k+cu\mu (\delta +\beta )}, & Y_{2}=\frac{b}{c}, & D_{2}=\frac{ab}{c(\beta +\delta )}, & V_{2}=\frac{ab\beta }{cu(\delta +\beta )},\\ Z_{2}=\frac{sack\beta e^{-\lambda \tau }-ab\beta \delta k-\mu c\delta u\beta -c\mu \delta^{2}u}{p(ab\beta \delta k+\mu c\delta u\beta +c\mu \delta u)}, & & & \end{array}$$

$$E_{3}=\left(X_{3},Y_{3},D_{3},V_{3},W_{3},0\right),$$

where

$$\begin{array}{c} X_{3}=\frac{sg}{kh+g\mu },Y_{3}=\frac{kshe^{-\lambda \tau }}{\delta (kh+g\mu )},D_{3}=\frac{akshe^{-\lambda \tau }}{\delta (\beta +\delta )(kh+g\mu )},V_{3}=\frac{h}{g},\\ W_{3}=\frac{sagk\beta e^{-\lambda \tau }-ag\beta \delta k-\mu h\delta u\beta -g\mu \delta^{2}u-hku\delta^{2}}{sq(\beta +\delta )(kh+g\mu )}, \end{array}$$

and

$$E_{4}=\left(X_{4},Y_{4},D_{4},V_{4},W_{4},Z_{4}\right),$$

where

$$\begin{array}{c} X_{4}=\frac{sg}{g\mu +kh},Y_{4}=\frac{b}{c},D_{4}=\frac{ab}{c(\beta +\delta )},V_{4}=\frac{h}{g},W_{4}=\frac{ab\beta g-chu(\beta +\delta )}{chq(\beta +\delta )},\\ Z_{4}=\frac{sckhe^{-\lambda \tau }-\delta \mu bg-\delta bkh}{bp(kh+g\mu )}. \end{array}$$

## 3. Mathematical Analysis of the Optimal Control

### 3.1. The Optimization Problem

To state the optimization problem, we first suppose that $\eta_{1}$ and $\eta_{2}$ vary in time. The problem (1) becomes then

(6) $$\left\{\begin{array}{c} \;\frac{dX}{dt}=s-\mu X\left(t\right)-k\left(1-\eta_{1}\left(t\right)\right)X\left(t\right)V\left(t\right),\\ \;\frac{dY}{dt}=e^{-\lambda \tau }k\left(1-\eta_{1}\left(t\right)\right)X\left(t-\tau \right)V\left(t-\tau \right)-\delta Y\left(t\right)-pY\left(t\right)Z\left(t\right),\\ \;\frac{dD}{dt}=\left(1-\eta_{2}\left(t\right)\right)aY\left(t\right)-\beta D\left(t\right)-\delta D\left(t\right),\\ \;\frac{dV}{dt}=\beta D\left(t\right)-uV\left(t\right)-qV\left(t\right)W\left(t\right),\\ \;\frac{dW}{dt}=gV\left(t\right)W\left(t\right)-hW\left(t\right),\\ \;\frac{dZ}{dt}=cY\left(t\right)Z\left(t\right)-bZ\left(t\right). \end{array}\right.$$

For this problem, we will have the following result of the boundedness and the positivity of any solutions:

**Theorem** **2.** *For any initial conditions X(θ), Y(θ), D(θ), V(θ), W(θ) and Z(θ)) verifying* (2) *and* (3), *the system* (6) *has a unique solution; moreover, this solution is non-negative and bounded for all t≥0.*

**Proof.**  Using the classical theory of the functional differential equations [23], it is clear to see that there is a unique local solution (X(t),Y(t),D(t),V(t),W(t),Z(t)) to system (1) in $\left[0,t_{m}\right)$.

From the system (6), we have

$$X\left(t\right)=e^{-\int_{0}^{t}\left(\mu +k\left(1-\eta_{1}\left(\xi \right)\right)V\left(\xi \right)\right)d\xi }\left(X\left(0\right)+se^{-\int_{0}^{t}(e^{-\int_{0}^{\eta }\mu +k\left(1-\eta_{1}\left(\xi \right)\right)V\left(\xi \right))d\xi }d\eta }\right),$$

$$\begin{array}{cc} Y(t)= & Y\left(0\right)e^{-\int_{0}^{t}\left(\delta +pZ\left(\xi \right)\right)d\xi }+e^{-\int_{0}^{t}\left(\delta +pZ\left(\xi \right)\right)d\xi }\\ & \times \int_{0}^{t}ke^{-\lambda \tau }\left(1-\eta_{1}\left(\zeta \right)\right)X\left(\zeta -\tau \right)V\left(\zeta -\tau \right)e^{\int_{0}^{\zeta }\left(\delta +pZ\left(\xi \right)\right)d\xi }d\zeta , \end{array}$$

$$D\left(t\right)=e^{-(\delta +\beta )t}\left(D\left(0\right)+\int_{0}^{t}\left(1-\eta_{2}\left(\eta \right)\right)aY\left(\eta \right)e^{(\delta +\beta )\eta }d\eta \right),$$

$$V\left(t\right)=e^{-\int_{0}^{t}\left(u+qW\left(\xi \right)\right)d\xi }\left(V\left(0\right)+\int_{0}^{t}\beta D\left(\eta \right)e^{\int_{0}^{\eta }\left(u+qW\left(\xi \right)\right)d\xi }d\eta \right),$$

$$W\left(t\right)=W\left(0\right)e^{\int_{0}^{t}\left(gV\left(\xi \right)-h\right)d\xi }$$

and

$$Z\left(t\right)=Z\left(0\right)e^{\int_{0}^{t}\left(cI\left(\xi \right)-b\right)d\xi },$$

from all these previous equalities, we deduce that all solutions are non-negative in $t\in [0,t_{m})$.

About the boundedness, we will prove that the solutions are bounded in each interval [nτ,(n+1)τ] such that n∈N.

We will begin with n=0. Let t∈[0,τ], from the first equation of system (6), we obtain

$$\frac{dX}{dt}\leq s-\mu X\left(t\right),$$

so,

$$X\left(t\right)\leq \left(X\left(0\right)-\frac{s}{\mu }\right)e^{-\mu t}+\frac{s}{\mu },$$

this means that *X* is bounded.

From the second equation of (6), we obtain

$$\frac{dY}{dt}\leq e^{-\lambda \tau }k\left(1-\eta_{1}\left(t\right)\right)V\left(t-\tau \right)X\left(t-\tau \right)-\delta I\left(t\right),$$

since $(1-\eta_{1}\left(t\right))\leq 1$ and $e^{-\lambda \tau }\leq 1$, it follows

$$\frac{dY}{dt}\leq kV\left(t-\tau \right)X\left(t-\tau \right)-\delta Y\left(t\right),$$

therefore,

$$Y\left(t\right)\leq Y\left(0\right)e^{-\delta t}+\int_{0}^{t}kV\left(\xi -\tau \right)X\left(\xi -\tau \right)e^{\delta (\xi -t)}d\xi ,$$

since (t−τ)∈[−τ,0] and from (2) and (3), we have the fact that V(t−τ)X(t−τ) is bounded, then *Y* is also bounded.

From the third equation of (6), we obtain

$$\frac{dD}{dt}=\left(1-\eta_{2}\left(t\right)\right)aY\left(t\right)-\beta D\left(t\right)-\delta D\left(t\right),$$

since $(1-\eta_{2}\left(t\right))\leq 1$, it follows

$$\frac{dD}{dt}\leq aY\left(t\right)-\beta D\left(t\right)-\delta D\left(t\right),$$

this inequality implies that

$$D\left(t\right)\leq D\left(0\right)e^{-(\delta +\beta )t}+\int_{0}^{t}aI\left(\xi \right)e^{\left(\delta +\beta )(\xi -t\right)}d\xi ,$$

from the boundedness result of *I*, one can conclude that *D* is bounded.

From the fourth equation of (6), we obtain

$$\frac{dV}{dt}\leq \beta D\left(t\right)-uV\left(t\right),$$

then,

$$V\left(t\right)\leq e^{-ut}V\left(0\right)+\int_{0}^{t}\beta D\left(\xi \right)e^{u(\xi -t)}d\xi ,$$

from the boundedness result of *D*, we conclude that *V* is also bounded.

From both the fourth and the fifth equations of (6), we obtain

$$\frac{dW}{dt}+hW\left(t\right)=gV\left(t\right)W\left(t\right)=\frac{g}{q}\left(\beta D\left(t\right)-uV\left(t\right)-\dot{V}\right),$$

then

$$\begin{array}{cc} W\left(t\right)\leq W\left(0\right)e^{-ht}+\frac{g}{q}( & \int_{0}^{t}\left(\beta D\left(\xi \right)+\left(h-u\right)V\left(\xi \right)\right)e^{h(\xi -t)}d\xi \\ & -V\left(t\right)+V\left(0\right)e^{-ht}), \end{array}$$

from the boundedness results of *D* and *V*, we deduce that *W* is bounded.

From the second and the last equation of system (6), we obtain

$$\begin{array}{cc} \frac{dZ}{dt}+bZ\left(t\right) & =cI(t)Z(t)\\ & =\frac{c}{p}\left(ke^{-\lambda \tau }\left(1-\eta_{1}\left(t\right)\right)V\left(t-\tau \right)H\left(t-\tau \right)-\delta I\left(t\right)-\dot{I}\right), \end{array}$$

then

$$\begin{array}{cc} Z\left(t\right)\leq Z\left(0\right)e^{-bt}+\frac{c}{p}( & \int_{0}^{t}\left(kV\left(\xi -\tau \right)X\left(\xi -\tau \right)+\left(b-\delta \right)Y\left(\xi \right)\right)e^{b(\xi -t)}d\xi \\ & -Y\left(t\right)+Y\left(0\right)e^{-bt}), \end{array}$$

from the boundedness results of *X*, *Y* and *V*, it follows the result that *Z* is bounded.

By following the same analysis as before, for each single interval [nτ,(n+1)τ] with n≥1, one can conclude that all the solutions are bounded for all t≥0. Therefore, every local solution can be prolonged up to any time $t_{m}>0$, which means that the solution exists globally. ☐

Let us consider the following objective functional:

(7) $$\begin{array}{c} J\left(\eta_{1},\eta_{2}\right)=\int_{0}^{t_{f}}\left\{X\left(t\right)+W\left(t\right)+Z\left(t\right)-\left[\frac{A_{1}}{2}\eta_{1}^{2}\left(t\right)+\frac{A_{2}}{2}\eta_{2}^{2}\left(t\right)\right]\right\}dt, \end{array}$$

where $t_{f}$ is the time period of therapy and the two positive constants $A_{1}$ and $A_{2}$ are based on the benefit-cost of the therapy $\eta_{1}$ and $\eta_{2}$, respectively. The two control functions, $\eta_{1}\left(t\right)$ and $\eta_{2}\left(t\right)$ are supposed to be bounded and also Lebesgue integrable. Our main purpose is to maximize the objective functional defined in the Equation (7) by maximizing the number of the uninfected cells, increasing the CTL immune responses and the antibodies, decreasing the viral load and also decreasing the cost of treatment. That means, we are seeking an optimal control pair $\left(\eta_{1}^{*},\eta_{2}^{*}\right)$ such that

(8) $$\begin{array}{c} J\left(\eta_{1}^{*},\eta_{2}^{*}\right)=max\left\{J\left(\eta_{1},\eta_{2}\right):\left(\eta_{1},\eta_{2}\right)\in U\right\}, \end{array}$$

where U is the control set given by

(9) $$\begin{array}{c} U=\{\left(\eta_{1}\left(t\right),\eta_{2}\left(t\right)\right):\eta_{i}\left(t\right)\;\mathrm{measurable},0\leq \eta_{i}\left(t\right)\leq 1,t\in \left[0,t_{f}\right],i=1,2\}. \end{array}$$

### 3.2. An Optimal Control Existence Result

The two optimal control pair existence result can be obtained via the results [24,25]. Indeed, we have the following result:

**Theorem** **3.** There exists an optimal control $(\eta_{1}^{*},\eta_{2}^{*})\in U$ such that

(10) $$\begin{array}{c} J\left(\eta_{1}^{*},\eta_{2}^{*}\right)=\max_{(\eta_{1},\eta_{2})\in U}J\left(\eta_{1},\eta_{2}\right). \end{array}$$

**Proof.**  To use the existence result [24], we should first check the following properties

(*C*_1_) The set of the corresponding state variables and controls is nonempty.
(*C*_2_) The set U is closed and convex.
(*C*_3_) The right hand side of the state system is bounded by a linear function in the state and control variables.
(*C*_4_) The integrand of the objective functional is concave on U.
(*C*_5_) There exists an α>1 and two constants $c_{1},c_{2}>0$, such that the integrand $I(X,W,Z,\eta_{1},\eta_{2})$ of the objective functional satisfies
  (11) $$\begin{array}{c} I\left(X,W,Z,\eta_{1},\eta_{2}\right)\leq c_{2}-c_{1}{\left(|\eta_{1}{|}^{2}+|\eta_{2}{|}^{2}\right)}^{{}^{\frac{\alpha }{2}}}, \end{array}$$
  where
  (12) $$\begin{array}{c} I\left(X,W,Z,\eta_{1},\eta_{2}\right)=X\left(t\right)+W\left(t\right)+Z\left(t\right)-\left[\frac{A_{1}}{2}\eta_{1}^{2}\left(t\right)+\frac{A_{2}}{2}\eta_{2}^{2}\left(t\right)\right]. \end{array}$$

The boundedness of the state system equations with the two controls (6) ensures us the existence of a solution. We can therefore deduce that the set of controls and the corresponding state variables are non-empty, this gives us the condition $\left(C_{1}\right)$. The control set is convex and closed by definition, which ensures the condition $\left(C_{2}\right)$. Moreover, since the system of state is bi-linear in $\eta_{1}$, $\eta_{2}$, the right hand-side of (6) verifies condition $\left(C_{3}\right)$, using the fact that the solutions are bounded. About the condition $\left(C_{4}\right)$, we obtain the Hessian matrix for *I* as follows,

(13) $$H_{I}=\left(\begin{array}{cc} -A_{1} & 0\\ 0 & -A_{2} \end{array}\right),$$

its determinant is stated as follows,

$$\begin{array}{c} det\left(H_{I}\right)=A_{1}A_{2}\geq 0,\;\forall \left(\eta_{1},\eta_{2}\right)\in U, \end{array}$$

then *I* is concave on U.

For the condition $\left(C_{5}\right)$, we have

(14) $$\begin{array}{c} I\left(X,W,Z,\eta_{1},\eta_{2}\right)\leq c_{2}-c_{1}\left(|\eta_{1}{|}^{2}+|\eta_{2}{|}^{2}\right), \end{array}$$

with $c_{2}$ depends on the upper bound on *X*, *W*, *Z*, and $c_{1}=min\left(\frac{A_{1}}{2},\frac{A_{2}}{2}\right)>0$. We deduce that there exists an optimal control pair $(\eta_{1}^{*},\eta_{2}^{*})\in U$ such that

$$\begin{array}{c} J\left(\eta_{1}^{*},\eta_{2}^{*}\right)=\max_{(\eta_{1},\eta_{2})\in U}J\left(\eta_{1},\eta_{2}\right). \end{array}$$

 ☐

### 3.3. The Optimality System

To prove the necessary conditions for the optimal control problem, we will use the Pontryagin’s minimum principle [26]. This principle changes (6), (7) and (9) into a problem of maximizing of an Hamiltonian, *T*, pointwise with respect to $\eta_{1}$ and $\eta_{2}$: $T\left(t,X,Y,D,V,W,Z,X_{\tau },V_{\tau },\eta_{1},\eta_{2},\lambda \right)=\frac{A_{1}}{2}\eta_{1}^{2}+\frac{A_{2}}{2}\eta_{2}^{2}-X-W-Z+\sum_{i=1}^{6}\lambda_{i}f_{i},$ where the $\lambda_{i}$ for i=1,…,6 is an adjoint variables and $f_{i}$ for i=1,…,6 is the system dynamics defined by

(15) $$\left\{\begin{array}{c} \;f_{1}=s-\mu X\left(t\right)-k\left(1-\eta_{1}\left(t\right)\right)X\left(t\right)V\left(t\right),\\ \;f_{2}=e^{-\lambda \tau }\left(1-\eta_{1}\left(t\right)\right)kH\left(t-\tau \right)V\left(t-\tau \right)-\delta Y\left(t\right)-pY\left(t\right)Z\left(t\right),\\ \;f_{3}=a\left(1-\eta_{2}\left(t\right)\right)Y\left(t\right)-\beta D\left(t\right)-\delta D\left(t\right),\\ \;f_{4}=\beta D\left(t\right)-uV\left(t\right)-qV\left(t\right)W\left(t\right),\\ \;f_{5}=gV\left(t\right)W\left(t\right)-hW\left(t\right),\\ \;f_{6}=cY\left(t\right)Z\left(t\right)-bZ\left(t\right), \end{array}\right.$$

By the Pontryagin’s minimum principle with the delay fact in state [26], we have the following theorem:

**Theorem** **4.** For any optimal control pair $\eta_{1}^{*},\eta_{2}^{*}$, and any solutions $\left(X^{*},Y^{*},D^{*},V^{*},W^{*},Z^{*}\right)$ (6), *there exists an adjoint variables, $\lambda_{1}$, $\lambda_{2}$, $\lambda_{3}$, $\lambda_{4}$,$\lambda_{5}$ and $\lambda_{6}$ satisfying*

(16) $$\left\{\begin{array}{cc} \lambda_{1}^{\prime }\left(t\right) & =1+\lambda_{1}\left(t\right)\left[\mu +k\left(1-\eta_{1}^{*}\left(t\right)\right)V^{*}\left(t\right)\right]\\ & +\chi_{\left[0,t_{f}-\tau \right]}\left(t\right)\lambda_{2}\left(t+\tau \right)\left(\eta_{1}^{*}\left(t+\tau \right)-1\right)ke^{-\lambda \tau }V^{*}\left(t\right),\\ \lambda_{2}^{\prime }\left(t\right) & =\lambda_{2}\left(t\right)\delta -\lambda_{3}\left(t\right)a\left(1-\eta_{2}^{*}\left(t\right)\right)-cZ^{*}\left(t\right)\lambda_{6}\left(t\right)+pZ^{*}\left(t\right)\lambda_{2}\left(t\right),\\ \lambda_{3}^{\prime }\left(t\right) & =\lambda_{3}\left(t\right)\left(\delta +\beta \right)-\beta \lambda_{4}\left(t\right)\\ \lambda_{4}^{\prime }\left(t\right) & =\lambda_{1}\left(t\right)\left[k\left(1-\eta_{1}^{*}\left(t\right)\right)X^{*}\left(t\right)\right]+\lambda_{4}\left(t\right)\left(u+qW^{*}\left(t\right)\right)\\ & +\chi_{\left[0,t_{f}-\tau \right]}\left(t\right)\lambda_{2}\left(t+\tau \right)\left[ke^{-\lambda \tau }\left(\eta_{1}^{*}\left(t+\tau \right)-1\right)X^{*}\left(t\right)\right],\\ \lambda_{5}^{\prime }\left(t\right) & =1+\lambda_{4}\left(t\right)qV^{*}\left(t\right)+\lambda_{5}\left(t\right)\left[h-cV^{*}\left(t\right)\right]\\ \lambda_{6}^{\prime }\left(t\right) & =1+\lambda_{2}\left(t\right)pY^{*}\left(t\right)+\lambda_{6}\left(t\right)\left[b-cY^{*}\left(t\right)\right] \end{array}\right.$$

*where the transversality conditions*

(17) $$\begin{array}{c} \lambda_{i}\left(t_{f}\right)=0,i=1,\ldots ,6. \end{array}$$

*Moreover, the optimal control as follows,*

(18) $$\begin{array}{cc} \eta_{1}^{*}= & min\left(1,max\left(0,\frac{k}{A_{1}}\left[\lambda_{2}\left(t\right)e^{-\lambda \tau }V_{\tau }^{*}X_{\tau }^{*}-\lambda_{1}\left(t\right)V^{*}\left(t\right)X^{*}\left(t\right)\right]\right)\right)\\ \eta_{2}^{*}= & min\left(1,max\left(0,\frac{1}{A_{2}}\lambda_{3}\left(t\right)aY^{*}\left(t\right)\right)\right). \end{array}$$

**Proof.**  The transversality conditions and adjoint equations as follows,

(19) $$\left\{\begin{array}{ccc} \; & \lambda_{1}^{\prime }\left(t\right)=-\frac{\partial T}{\partial X}\left(t\right)-\chi_{\left[0,t_{f}-\tau \right]}\left(t\right)\frac{\partial T}{\partial X_{\tau }}\left(t+\tau \right),\; & \lambda_{1}\left(t_{f}\right)=0,\\ \; & \lambda_{2}^{\prime }\left(t\right)=-\frac{\partial T}{\partial Y}\left(t\right),\; & \lambda_{2}\left(t_{f}\right)=0,\\ \; & \lambda_{3}^{\prime }\left(t\right)=-\frac{\partial T}{\partial D}\left(t\right),\; & \lambda_{3}\left(t_{f}\right)=0,\\ \; & \lambda_{4}^{\prime }\left(t\right)=-\frac{\partial T}{\partial V}\left(t\right)-\chi_{\left[0,t_{f}-\tau \right]}\left(t\right)\frac{\partial T}{\partial V_{\tau }}\left(t+\tau \right),\; & \lambda_{4}\left(t_{f}\right)=0,\\ \; & \lambda_{5}^{\prime }\left(t\right)=-\frac{\partial T}{\partial W}\left(t\right),\; & \lambda_{5}\left(t_{f}\right)=0.\\ \; & \lambda_{6}^{\prime }\left(t\right)=-\frac{\partial T}{\partial Z}\left(t\right),\; & \lambda_{6}\left(t_{f}\right)=0. \end{array}\right.$$

The two optimal controls $\eta_{1}^{*}$ and $\eta_{2}^{*}$ can be solved from the optimality conditions,

$$\begin{array}{c} \frac{\partial T}{\partial \eta_{1}}\left(t\right)=0,\;\mathrm{at}\;\eta_{2}^{*},\;\frac{\partial T}{\partial \eta_{2}}\left(t\right)=0,\;\mathrm{at}\;\eta_{2}^{*}. \end{array}$$

$$\begin{array}{cc} \frac{\partial T}{\partial \eta_{1}}\left(t\right)= & A_{1}\eta_{1}\left(t\right)+k\lambda_{1}\left(t\right)v\left(t\right)X\left(t\right)-k\lambda_{2}\left(t\right)V_{\tau }X_{\tau }e^{-\lambda \tau }=0,\\ \frac{\partial T}{\partial \eta_{2}}\left(t\right)= & A_{2}\eta_{2}\left(t\right)-a\lambda_{3}\left(t\right)Y\left(t\right)=0. \end{array}$$

By the definition of U, we obtain

$$\begin{array}{cc} \eta_{1}^{*}= & min\left(1,max\left(0,\frac{k}{A_{1}}\left[\lambda_{2}\left(t\right)e^{-\lambda \tau }V_{\tau }^{*}X_{\tau }^{*}-\lambda_{1}\left(t\right)V^{*}\left(t\right)X^{*}\left(t\right)\right]\right)\right)\\ \eta_{2}^{*}= & min\left(1,max\left(0,\frac{1}{A_{2}}\lambda_{3}\left(t\right)aY^{*}\left(t\right)\right)\right). \end{array}$$

If we replace $\eta_{1}^{*}$ and $\eta_{2}^{*}$ in the systems (6), we have the following optimality system:

$$\begin{array}{cc} \frac{dX^{*}}{dt} & =s-\mu X^{*}\left(t\right)-k\left(1-\eta_{1}^{*}\left(t\right)\right)V^{*}\left(t\right)X^{*}\left(t\right),\\ \frac{dY^{*}}{dt} & =e^{-\lambda \tau }k\left(1-\eta_{1}^{*}\left(t\right)\right)V^{*}\left(t-\tau \right)X^{*}\left(t-\tau \right)-\delta Y^{*}\left(t\right)-pY^{*}\left(t\right)Z^{*}\left(t\right),\\ \frac{dD^{*}}{dt} & =\left(1-\eta_{2}^{*}\left(t\right)\right)aY^{*}\left(t\right)-\delta D^{*}\left(t\right)-\beta D^{*}\left(t\right)\\ \frac{dV^{*}}{dt} & =\beta D^{*}\left(t\right)-uV^{*}\left(t\right)-qV^{*}\left(t\right)W^{*}\left(t\right),\\ \frac{dW^{*}}{dt} & =gV^{*}\left(t\right)W^{*}\left(t\right)-hW^{*}\left(t\right),\\ \frac{dZ^{*}}{dt} & =cY^{*}\left(t\right)Z^{*}\left(t\right)-bZ^{*}\left(t\right), \end{array}$$

then,

(20) $$\left\{\begin{array}{cc} \lambda_{1}^{\prime }\left(t\right) & =1+\lambda_{1}\left(t\right)\left[\mu +k\left(1-\eta_{1}^{*}\left(t\right)\right)V^{*}\left(t\right)\right]\\ & +\chi_{\left[0,t_{f}-\tau \right]}\left(t\right)\lambda_{2}\left(t+\tau \right)\left(\eta_{1}^{*}\left(t+\tau \right)-1\right)ke^{-\lambda \tau }V^{*}\left(t\right),\\ \lambda_{2}^{\prime }\left(t\right) & =\lambda_{2}\left(t\right)\delta -\lambda_{3}\left(t\right)a\left(1-\eta_{2}^{*}\left(t\right)\right)-cZ^{*}\left(t\right)\lambda_{6}\left(t\right)+pZ^{*}\left(t\right)\lambda_{2}\left(t\right),\\ \lambda_{3}^{\prime }\left(t\right) & =\lambda_{3}\left(t\right)\left(\delta +\beta \right)-\beta \lambda_{4}\left(t\right)\\ \lambda_{4}^{\prime }\left(t\right) & =\lambda_{1}\left(t\right)\left[k\left(1-\eta_{1}^{*}\left(t\right)\right)X^{*}\left(t\right)\right]+\lambda_{4}\left(t\right)\left(u+qW^{*}\left(t\right)\right)\\ & +\chi_{\left[0,t_{f}-\tau \right]}\left(t\right)\lambda_{2}\left(t+\tau \right)\left[ke^{-\lambda \tau }\left(\eta_{1}^{*}\left(t+\tau \right)-1\right)X^{*}\left(t\right)\right],\\ \lambda_{5}^{\prime }\left(t\right) & =1+\lambda_{4}\left(t\right)qV^{*}\left(t\right)+\lambda_{5}\left(t\right)\left[h-cV^{*}\left(t\right)\right]\\ \lambda_{6}^{\prime }\left(t\right) & =1+\lambda_{2}\left(t\right)pY^{*}\left(t\right)+\lambda_{6}\left(t\right)\left[b-cY^{*}\left(t\right)\right] \end{array}\right.$$

(21) $$\begin{array}{c} \lambda_{i}\left(t_{f}\right)=0,i=1,\ldots ,6. \end{array}$$

 ☐

## 4. Numerical Results

To illustrate the numerical simulations, we implement and solved numerically our optimality system by the finite difference approximation method [27,28,29]. We obtain the following algorithm (Algorithm 1):   
**Algorithm 1:** The forward-backward finite difference numerical scheme.Step 1:for j=−M,…,0, do:     $X_{j}=X_{0}$, $Y_{j}=Y_{0}$, $D_{j}=D_{0}$, $V_{j}=V_{0}$, $Z_{j}=Z_{0}$, $\eta_{1}^{j}=0$, $\eta_{2}^{j}=0$.end forfor j=N,…,N+M, do:     $\lambda_{1}^{j}=0,\lambda_{2}^{j}=0,\lambda_{3}^{j}=0,\lambda_{4}^{j}=0,\lambda_{5}^{j}=0,\lambda_{6}^{j}=0.$end forStep 2:for j=0,…,N−1, do:     $X_{j+1}=X_{j}+h\left[s-\mu X_{j}-k\left(1-\eta_{1}^{j}\right)V_{j}X_{j}\right],$     $Y_{j+1}=Y_{j}+h\left[ke^{-\lambda \tau }\left(1-\eta_{1}^{j}\right)X_{j-M}V_{j-M}-\delta Y_{j}-pY_{j}Z_{j}\right],$     $D_{j+1}=D_{j}+h\left[\left(1-\eta_{2}^{i}\right)aY_{j}-\delta D_{j}-\beta D_{j}\right],$     $V_{j+1}=V_{j}+h\left[\beta D_{j}-uV_{j}-qV_{j}W_{j}\right],$     $W_{j+1}=W_{j}+h\left[gV_{j}W_{j}-hW_{j}\right],$     $Z_{j+1}=Z_{j}+h\left[cY_{j}Z_{j}-bZ_{j}\right],$     $\lambda_{1}^{N-j-1}=\lambda_{1}^{N-j}-h\left[1+\lambda_{1}^{N-j}\left(\mu +k\left(1-\eta_{1}^{j}\right)V_{j+1}\right)\right]$                $+\chi_{\left[0,t_{f}-\tau \right]}\left(t_{N-j}\right)\lambda_{2}^{N-j+M}k\left(\eta_{1}^{j+M}-1\right)e^{-\lambda \tau }V_{j+1}],$     $\lambda_{2}^{N-j-1}=\lambda_{2}^{N-j}-h\left[\lambda_{2}^{N-j}\left(\delta +pZ_{j+1}\right)-\lambda_{3}^{N-j}a\left(1-\eta_{2}^{j}\right)-\lambda_{6}^{N-j}cZ_{j+1}\right],$     $\lambda_{3}^{N-j-1}=\lambda_{3}^{N-j}-h\left[\lambda_{3}^{N-j}\left(\delta +\beta \right)-\lambda_{4}^{N-j}\beta \right],$     $\lambda_{4}^{N-j-1}=\lambda_{4}^{N-j}-h[\lambda_{1}^{N-j}k\left(1-\eta_{1}^{j}\right)X_{j+1}+\lambda_{4}^{N-j}\left(u+qW_{j+1}\right)$                $+\chi_{\left[0,t_{f}-\tau \right]}\left(t_{N-j}\right)\lambda_{2}^{N-j+M}k\left(\eta_{1}^{j+M}-1\right)e^{-\lambda \tau }X_{j+1}],$     $\lambda_{5}^{N-j-1}=\lambda_{5}^{N-j}-h\left[1+\lambda_{2}^{N-j}qV_{j+1}+\lambda_{5}^{N-j}\left(h-gV_{j+1}\right)\right],$     $\lambda_{6}^{N-j-1}=\lambda_{6}^{N-j}-h\left[1+\lambda_{2}^{N-j}pY_{j+1}+\lambda_{6}^{N-j}\left(b-cY_{j+1}\right)\right],$     $R_{1}^{j+1}=\left(1/A_{1}\right)\left(k\lambda_{2}^{N-j-1}e^{-\lambda \tau }V_{j-M+1}X_{j-M+1}-k\lambda_{1}^{N-j-1}V_{j+1}X_{j+1}\right)$     $R_{2}^{j+1}=\left(1/A_{2}\right)\lambda_{3}^{N-j-1}aY_{j+1},$     $\eta_{1}^{j+1}=min\left(1,max\left(R_{1}^{j+1},0\right)\right),$     $\eta_{2}^{j+1}=min\left(1,max\left(R_{2}^{j+1},0\right)\right),$end forStep 3:for j=1,…,N, write  $X^{*}\left(t_{j}\right)=X_{j},Y^{*}\left(t_{j}\right)=Y_{j},D^{*}\left(t_{j}\right)=D_{j},V^{*}\left(t_{j}\right)=V_{j},W^{*}\left(t_{j}\right)=W_{j}$, $Z^{*}\left(t_{j}\right)=Z_{j},\eta_{1}^{*}\left(t_{j}\right)=\eta_{1}^{j},\eta_{2}^{*}\left(t_{j}\right)=\eta_{2}^{j}.$end for

Using values of parameters from [12,17]; i.e., $s=2.6\times 10^{7}$, $k=1.67\times 10^{-12}$, μ=0.01, δ=0.053, a=150, β=0.87, u=3.8, τ=5, $\lambda =1.1\times 10^{-2}$, $q=10^{-12}$, $g=10^{-4}$, h=0.1, p=0.01, b=0.2, c=0.03, $A_{1}=50,000$ and $A_{2}=5000$. The role of the two parameters $A_{1}$ and $A_{2}$ is to calibrate the terms size in the system equations.

**Figure 1:**
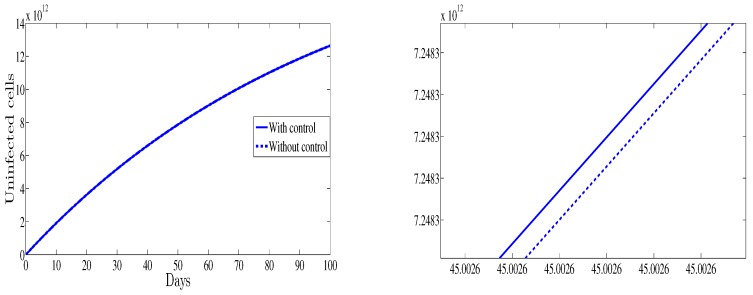
The evolution of the healthy cells (**left**) vs. time and a zoomed in region (**right**).

Figure 1 depicts the evolution of the uninfected cells as function of time for both cases with and without control therapy. It is shown that with control the number of the uninfected cells is higher than those observed for the case without control. This result support the fact that the control strategy is to maximize the number of the healthy cells.

**Figure 2:**
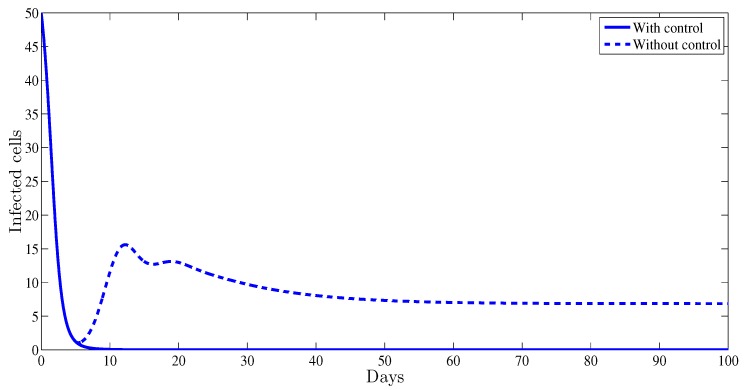
The evolution of the hepatitis B virus (HBV) infected cells vs. time.

From Figure 2, one can observe that the plot representing the infected cells with control strategy converges towards 0.002, however without any control therapy it converges towards 7.69, which proves that administrating this treatment will help the patient by a significant reduction of the infected cells.

**Figure 3:**
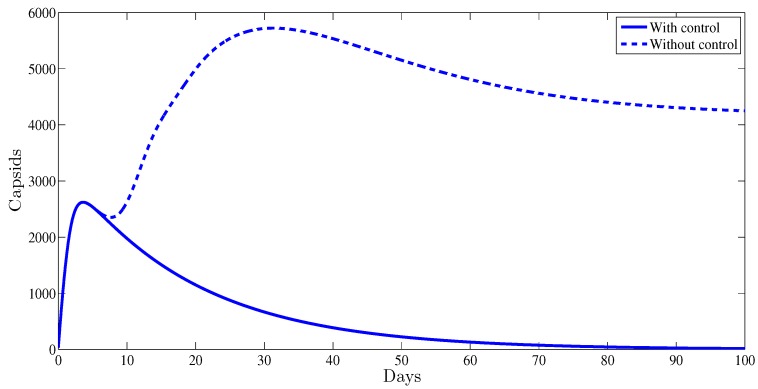
The evolution of HBV capsids vs. time.

Figure 3 illustrates the evolution of the capsids during the period of observation. It was established that with a control strategy, the amount of capsids vanishes after the first weeks of the administrated therapy. Meanwhile, without any control strategy this number remains at very high positive level, $4.37\times 10^{3}$.

**Figure 4:**
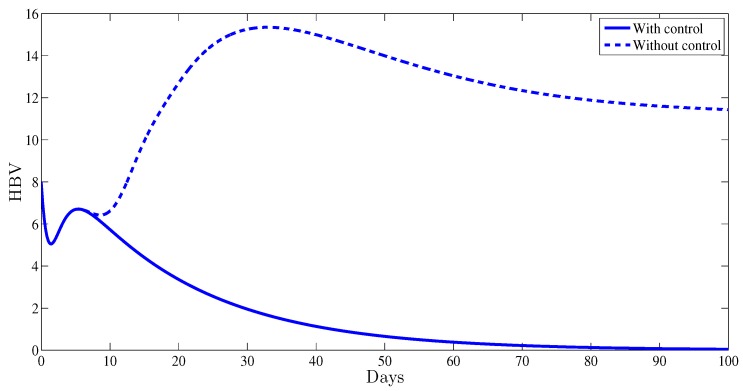
The free HBV virions as function of time.

The role of the two administrated therapy controls is also remarked in Figure 4. It was shown that with therapy control, the number of virions dies out after the first weeks of therapy, while without any control strategy it remains equal to 11.53. This indicates clearly the impact of the administrated therapy in controlling the HBV viral replication.

**Figure 5:**
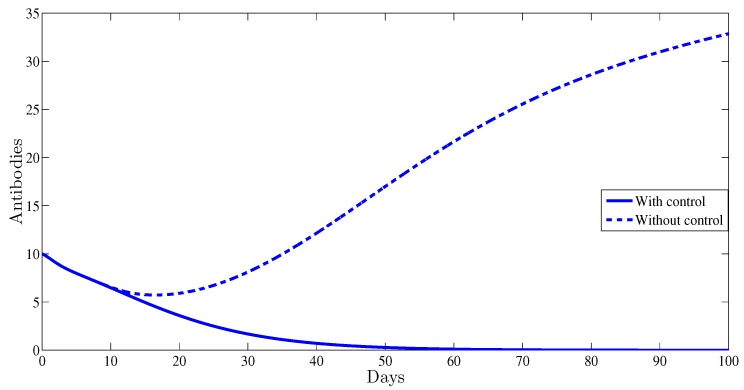
The evolution of antibodies as function of time.

The antibody immune response is clearly affected by the control. This is illustrated in Figure 5; indeed, with control, the curve of antibodies converges towards zero; however, without any control strategy it converges towards 33.09 which clearly indicates the importance of adding the antibody component to HBV viral dynamics.

**Figure 6:**
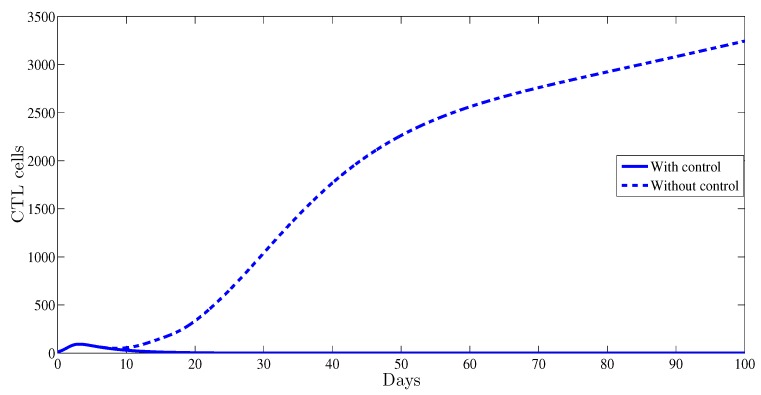
The behavior of the cytotoxic T-lymphocyte (CTL) immune response as function of time.

The CTL cell dynamics are also affected by the optimal control. This is shown in Figure 6; indeed, with control, the curve of CTL cells converges towards zero; however, without any control it converges towards a high level of $3.41\times 10^{3}$; this reveals the role of the CTL component in blocking HBV viral infection.

**Figure 7:**
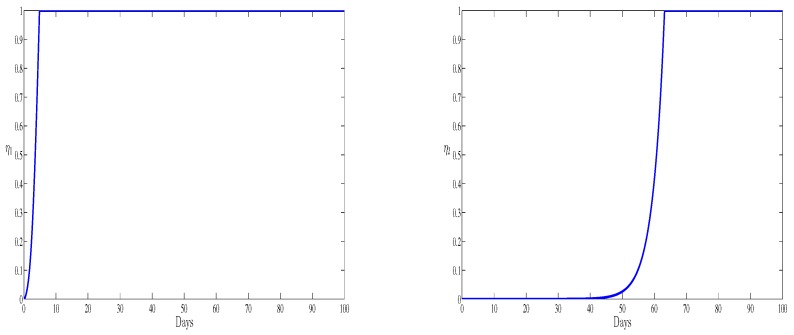
The optimal control $\eta_{1}$ (**left**) and the optimal control $\eta_{2}$ (**right**) versus time.

The two optimal controls $\eta_{1}$ and $\eta_{2}$ representing inhibiting new infections and blocking viral replication are represented in Figure 7. The two curves present the treatment administration schedule for the period of observation. Both of the controls start from zero and when the first immune boosting drug is administered at full scale, the second drug is at its lowest for the first days of observation. During the last days of observation both the therapies should be administrated at their full scales. In this case the new infection is totally blocked. It is worthy to notice that if we compare the numerical simulation of this paper and the recent work [17], we remark that the antibodies have a clear effect in maximizing the level of the healthy cells and reducing the amount of the free viruses. Also, we remark that the presence of antibodies improves the effectiveness of both treatments after the first 60 days by reaching their maximum value. However, without antibodies, only the second treatment presents efficacy (see [17]) which leads to the elimination of HBV viruses. The obtained numerical results show that with antibodies and the optimal control, we observe a significant reduce of the HBV infection. All our numerical simulations will performed during the acute HBV infection period. This period is also known as the early stage of the infection [12,30]; however it will be very useful to predict infection for a chronic type of the infection.

## 5. Conclusions

We have modeled HBV in regards to intracellular behavior, capsids and adaptive immunity. The considered adaptive immune system is represented by the cytotoxic T-lymphocyte cells and the antibodies. The model under consideration includes six nonlinear differential equations that describe the dynamics that occur between hepatitis B free viruses (HBV), HBV DNA-containing capsids, hepatocytes, the antibodies and the CTL cells. An intracellular infection time delay and the effect of two drugs are incorporated into the suggested model. We have established the existence and uniqueness of the optimal controls via Pontryagin’s maximum principle. The problem was implemented and solved numerically using backward and forward finite numerical difference schemes. It was established that with the two administrated optimal therapies, the amount of the healthy hepatocytes increases considerably while the number of infected hepatocytes decreases remarkably. Moreover, it was also shown that, with the control strategy, the viral load decreases significantly comparing with the model without control case, and this may boost the patient’s life quality. Finally, we would like to mention that the used optimal controllers are given by open-loop, it will be useful to test other feedback control methods as a new predictive control model.
